# Posterior reversible encephalopathy syndrome in a patient with late postpartum eclampsia

**DOI:** 10.1097/MD.0000000000035867

**Published:** 2023-11-10

**Authors:** Manmin Zhu, Hao Huang

**Affiliations:** a Department of Neurology, Affiliated Hospital of Zunyi Medical University, Zunyi, China.

**Keywords:** late postpartum eclampsia, posterior reversible encephalopathy syndrome, pregnancy

## Abstract

**Rationale:**

Posterior reversible encephalopathy syndrome (PRES) is a rare complication commonly associated with headache and acute changes in blood pressure that results from a variety of causes, culminating in vasogenic cerebral edema in the occipital and parietal lobes of the brain.

**Patient concerns:**

We report here a woman who suffered from headache, generalized tonic-clonic seizures, and cortical blindness in the late postpartum period.

**Diagnoses:**

Posterior reversible encephalopathy syndrome.

**Interventions:**

The patient was treated with amlodipine besylate tablets for hypertension, dehydration with mannitol and glycerin fructose, and antispasmodic treatment with sodium valproate and oxcarbazepine.

**Outcomes:**

On day 2, the patient became conscious, headache and vision improved. One week later, symptoms and signs disappeared, blood pressure returned to normal, and brain MRI lesions disappeared in re-examination.

**Lessons:**

Eclampsia associated with PRES is reversible in most cases, but it is a serious and potentially life-threatening obstetric emergency. If adequate treatment is provided in a timely manner, most women will make a full recovery. Attention needs to be paid to timely and adequate treatment, as well as appropriate follow-up and support for patients with PRES.

## 1. Introduction

Post-introduction reversible encephalopathy syndrome (PRES) is characterized by seizures, disturbance of consciousness, headache, visual symptoms, nausea/vomiting, and focal nervous system signs. PRES may be associated with a number of diseases, including hypertension, eclampsia, and autoimmune diseases. Among them, hypertension is the most common, and moderate to severe hypertension is observed in about 70% to 80% of patients.^[1]^ All of these lead to cerebrovascular edema, which seems to be the key pathogenic mechanism and as the name suggests, once the root cause is eliminated, it’s usually reversible but if treatment is delayed, PRES can lead to cerebral infarction and bleeding so it’s important to identify and treat PRES early and we reported one case A 36-year-old woman developed late postpartum eclampsia 5 days after delivery and was diagnosed with PRES by magnetic resonance imaging (MRI). After treatment, her clinical symptoms and imaging findings fully recovered.

## 2. Case report

**Figure 1.**
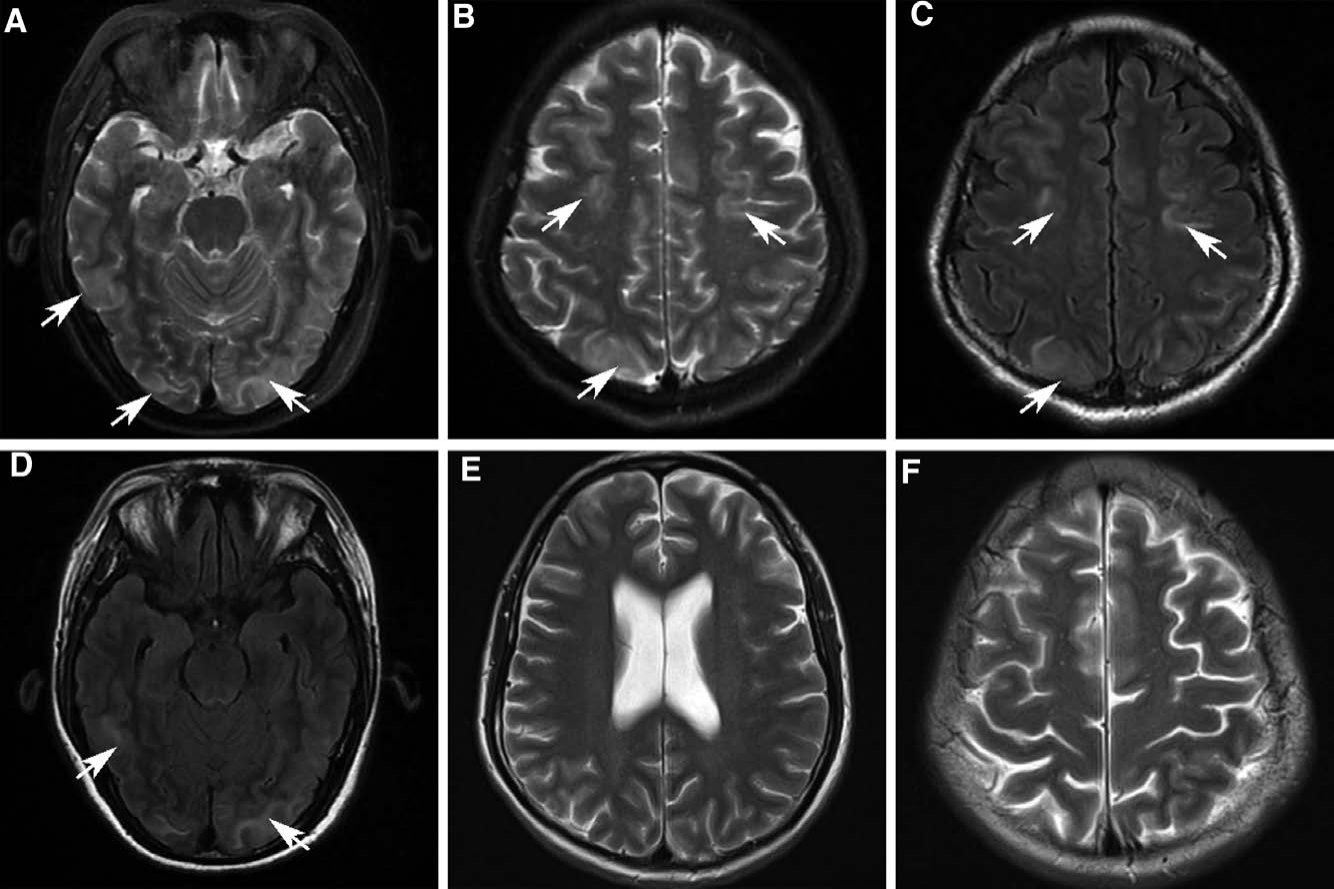
(A–D) Symmetrical patchy long T2 signals were observed in the subcortical regions of the occipital frontal lobes bilaterally; small patchy short T2 signals were observed in the posterior horns of the ventricles bilaterally. (E and F) No abnormal signal was found in the brain parenchyma area, and the boundary of gray matter was clear; the size and signal of ventricle system were not abnormal; the width and signal of cerebral sulci and cisterna were not abnormal.

A 36-year-old woman was admitted to the hospital on the 5th day after cesarean section due to recurrent headache for 6 days, exacerbation accompanied by disturbance of consciousness and limb twitching for 1 day. She had no history of eclampsia, normal prenatal examination and delivery records, and no neurological diseases. Physical examination: T 36.5°C, BP 166/65 mmHg. Physical examination of nervous system: lethargy, slow reaction, strong neck, decreased visual acuity in both eyes at about 3 horizontal fingers from neck and chin, unable to distinguish the fingers at 20 cm in front, symmetric tendon reflex (++) bilateral pupil is round and large, pupil diameter is about 3.0 mm, sensitive to light reflex directly and indirectly, muscle tension of limbs is normal, Babinski (−). Auxiliary examination: routine hematuria myocardial enzyme, liver and kidney function, coagulation function, antinuclear antibody, rheumatoid factor virus, no obvious abnormalities were found. The cerebral pressure was 210 mm H_2_O, and the biochemical indication of protein in cerebrospinal fluid was slightly elevated (49.1 mg/dL). Head MRI plain scan + Diffusion Weighted Imaging (DWI) + Susceptibility Weighted Imaging showed: symmetrical abnormal signals in the subcortical areas of the occipital and frontal lobes of both parietal lobes, considering the syndrome of reversible posterior encephalopathy (Fig. 1A–D).

Combined with the medical history, physical examination and auxiliary examination, she was diagnosed with post-introduction reversible encephalopathy syndrome. The patient was treated with amlodipine besylate tablets for hypertension, dehydration with mannitol and glycerin fructose, and antispasmodic treatment with sodium valproate and oxcarbazepine. On day 2, the patient became conscious, headache and vision improved. One week later, symptoms and signs disappeared, blood pressure returned to normal, and brain MRI lesions disappeared in reexamination (Fig. 1E and F).

## 3. Conclusions

PRES is an acute encephalopathy syndrome with headache, visual disturbance, seizure and consciousness disturbance as the main manifestations. Because the clinical presentation of PRES is usually nonspecific and onset is rapid (usually 12–24 hours), early recognition is often difficult and treatment is delayed. At present, there are 3 theories about the pathogenesis of PRES: hypoperfusion of the brain, cerebral vasospasm, blood brain barrier or capillary endothelial cells in the brain were damaged.^[2]^ Although the typical pathological changes of PRES were reversible vasogenic edema, the disease may develop further and result in cerebral infarction, cerebral hemorrhage and fatal hernia if there is a delay in the diagnosis and treatment of PRES.^[2,3]^

Imaging features are one of the core conditions for PRES diagnosis. MRI showed that T1-weighted images were mostly equal signal or low signal, T2-weighted images were high signal, fluid attenuated inversion recovery showed high signal change, DWI showed equal signal or low signal, apparent diffusion coefficient (ADC) showed high signal. MRI can clearly show angiogenic edema, especially T2 and fluid attenuated inversion recovery sequences.^[4]^ The typical imaging manifestations of PRES were subcortical cerebral edema, which was most common in parietal and occipital lobes, followed by frontal lobe, temporal lobe and cerebellar hemisphere. Atypical lesions include basal ganglia, brainstem, and deep white matter and may be unilateral or asymmetric.^[5]^ This disease is easily confused with cerebral ischemic stroke, which is cytotoxic cerebral edema. In the acute phase, cerebral ischemic stroke presents high signal on DWI and low signal on ADC. While PRES is vasogenic edema, most patients present equal signal or low signal on DWI and high signal on ADC.^[4,6]^ Although radiographic features are often used as a diagnostic tool, it is important to note that neither the type nor severity of cerebral edema was associated with the type or severity of clinical manifestations, according to a retrospective study.^[7]^ The symptoms and signs are nonspecific. The main purpose of brain imaging is to rule out other diagnoses. Therefore, the diagnosis of PRES is not mainly radiological, the clinical context and the judgment of the clinician are also crucial to making the correct diagnosis.

PRES can be induced by a variety of diseases and drugs, including hypertension, eclampsia, kidney failure, liver failure, autoimmune diseases, severe infections, organ transplantation, and immunosuppressants. Hypertension is the most common, and moderate to severe hypertension is observed in approximately 70% to 80% of patients.^[1]^ Hypertension during pregnancy is one of the leading causes of maternal death worldwide.^[8]^ Eclampsia is the most severe stage of hypertension in pregnancy and includes eclampsia before, during and after delivery. Late postpartum eclampsia refers to eclampsia that develops 2 days after delivery. Severe headaches and high blood pressure are the most common symptoms, along with edema, vision impairment, pain in the upper abdomen, seize-like seizures and other symptoms.^[9]^ In a retrospective study in Japan, PRES was present in approximately 20% of eclampsia patients with neurological symptoms.^[10]^

The key of PRES treatment is anti-cerebral edema treatment and active treatment for the primary disease. Early correction of elevated blood pressure and treatment of seizures are cornerstones of PRES treatment. In patients with acute hypertension, gradual reduction of blood pressure should be performed (no more than 20%–25% in the first few hours). The goal is to maintain mean arterial pressure between 105 and 125 mm Hg.^[11]^ At present, there is no unified standard of antiepileptic treatment for patients with PRES, and antiepileptic drugs must be used according to individual situations. Magnesium sulfate is indicated for the prevention of epileptic seizures in pregnant women. It has the effect of cerebral vasodilation and reduces blood vessel permeability. Most patients with PRES do not require long-term antiepileptic treatment. When symptoms are alleviated and risk factors are under control, drug reduction and discontinuation should be carried out in time.^[11]^

Eclampsia associated with PRES is reversible in most cases, but it is a serious and potentially life-threatening obstetric emergency. If adequate treatment is provided in a timely manner, most women will make a full recovery, with complete clinical and radiological improvement. Conversely, delayed treatment can lead to permanent disability or even death.^[3,12]^

Clinical and radiological findings of PRES have traditionally been considered reversible, but this has not been the case always. As mentioned earlier, cerebral hemorrhage and cerebral infarction are the most common causes of incomplete recovery, and in severe cases PRES may even be fatal. Severe complications highlight the need for timely identification and treatment of PRES etiology. Attention needs to be paid to timely and adequate treatment, as well as appropriate follow-up and support for patients with PRES.

## Author contributions

**Conceptualization:** Hao Huang.

**Data curation:** Manmin Zhu.

**Writing – original draft:** Manmin Zhu.

**Writing – review & editing:** Manmin Zhu.
